# Levels, Toxic Effects, and Risk Assessment of Pyrrolizidine Alkaloids in Foods: A Review

**DOI:** 10.3390/foods13040536

**Published:** 2024-02-09

**Authors:** Yu-Shun Lu, Jing Qiu, Xi-Yan Mu, Yong-Zhong Qian, Lu Chen

**Affiliations:** 1 Key Laboratory of Agro-Product Quality and Safety, Institute of Quality Standards and Testing Technology for Agro-Products, Chinese Academy of Agricultural Sciences, Beijing 100081, China; luyushun@caas.cn (Y.-S.L.); qianyongzhong@caas.cn (Y.-Z.Q.); 2 Institute of Special Animal and Plant Sciences, Chinese Academy of Agricultural Sciences, Changchun 130112, China

**Keywords:** pyrrolizidine alkaloids, food, PAs levels, hepatotoxicity, cytotoxicity, risk assessment

## Abstract

Pyrrolizidine alkaloids (PAs) are naturally occurring secondary metabolites of plants. To date, more than 660 types of PAs have been identified from an estimated 6000 plants, and approximately 120 of these PAs are hepatotoxic. As a result of PAs being found in spices, herbal teas, honey, and milk, PAs are considered contaminants in foods, posing a potential risk to human health. Here, we summarize the chemical structure, toxic effects, levels, and regulation of PAs in different countries to provide a better understanding of their toxicity and risk assessment. With recent research on the risk assessment of PAs, this review also discusses the challenges facing this field, aiming to provide a scientific basis for PA toxicity research and safety assessment.

## 1. Introduction

Plants and plant-derived products may contain naturally occurring toxins that can be harmful to humans. Pyrrolizidine alkaloids (PAs) are a class of natural toxins that have drawn increased amounts of attention [1]. PAs are secondary metabolites of plants produced by a defense mechanism against insects. To date, more than 600 PAs and their *N*-oxides (PANOs) have been identified from nearly 6000 plant species [2]. PAs are widely present in various plant-derived foods, such as spices, honey, and herbal teas, and can potentially pose a risk to human health via dietary intake [3]. Among these PAs, 1,2-unsaturated PAs have been proven to be carcinogenic and hepatotoxic to humans [3]. After metabolic (oxidative) activation of PAs into dehydropyrrolizidine (DHP) esters, adducts with DNA are formed and considered the major cause of the carcinogenic effects of PAs [4]. Incidents of liver damage caused by the consumption of PAs in foods have been reported [5] and are considered to be one of the major causes of hepatic venous occlusion disease (HVOD), which can lead to cirrhosis and liver failure. Long-term exposure to these pollutants has been associated with genotoxic and carcinogenic effects [1].

Due to the widespread presence of PAs/PANOs in different types of food, their presence in food should be recognized as a food safety issue. An assessment of PA/PANO levels during food processing can provide a realistic picture of PA exposure. For example, Casado et al. (2023) summarized the effects of heat treatment, fermentation, infusion preparation, milling, washing, and soaking on PAs during food processing [6]. Additionally, the most relevant analytical procedures for their determination in different food products were included from 2010 to 2020. The development of sensitive analytical methods can lead to a better understanding of PA occurrence [7]. However, it is important to note that the lack of sufficient toxicological data on PAs hinders their risk assessment. In addition, due to insufficient toxicity data on PAs, the toxic levels of PAs vary widely, and species vary widely in their sensitivity to PA exposure. Moreover, the risk assessment standards and legislation for PAs vary among different countries. Within the framework of current risk assessments, individual PAs are considered a group of equipotent substances with carcinogenic effects [8]. The large number of PAs/PANOs occurring in plants makes it impossible to generate comprehensive in vivo data on toxicity. Merz and Schrenk (2016) reported interim relative potency (REP) factors for the toxic and genotoxic potency of 1,2-unsaturated PAs based on limited cytotoxicity data [9]. Nevertheless, there is an urgent need for congener-specific data on relative toxicity and the use of PBPK modeling and structure–activity relationship considerations to better understand PA toxicity and genotoxicity. Therefore, the risk of PAs to human health cannot be ignored, and this food safety issue should be addressed. In this review, we performed a comprehensive survey on the occurrence of PAs in various food products from a global view and summarized the main types of PAs and their concentrations in specific foods based on publications from the last decade (2011–2023). Moreover, this review provides advanced knowledge about the in vitro toxicology of PAs for PBK modeling. Such information is highly important for in vitro alternative toxicology, i.e., computerized toxicology, for the risk assessment of PAs and therefore may contribute to the refinement of risk management and maximum residue limit (MRL) setting. In addition, the challenges associated with PA risk assessment in foods will be discussed.

## 2. Chemical Structure and Toxicity of PAs

**Figure 1:**
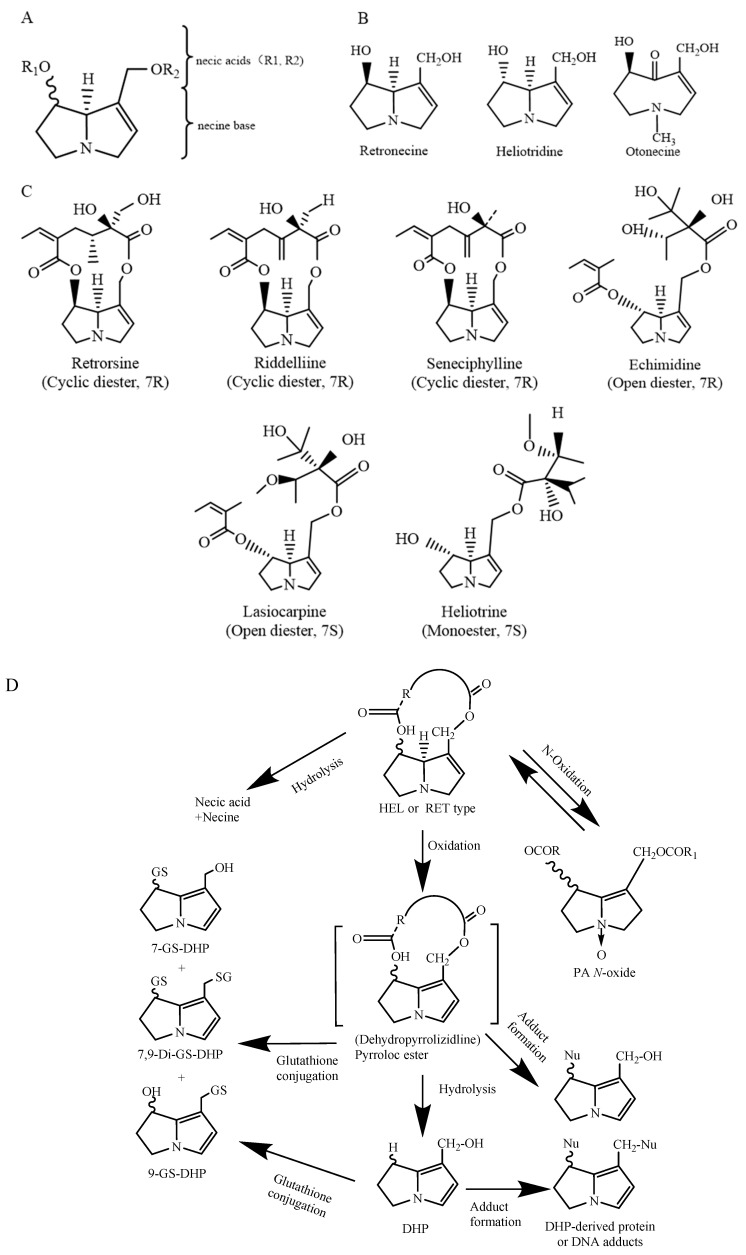
The chemical structure of 1,2-unsaturated PAs (**A**); retronecine (RET) type, heliotrine (HEL) type, and otonecine (OTO) type PAs; schematic diagram (**B**); chemical structural formulas of typical RET- and HEL-type PAs (**C**). Modified from [10]; heliotridine or retronecine-type PA metabolism [4,11] (**D**).

PAs are mostly formed by a pyrrolizidine ring and an esterified organic acid, with the pyrrolizidine ring referred to as the necine and the acid part as the necic acid (Figure 1A). Based on the presence or absence of unsaturated double bonds at positions C-1 and C-2 of the necine structure, PAs are divided into saturated and unsaturated types. Saturated PAs with a saturated necine base, such as the platynecine (PLA) type, are known to be non-toxic. The unsaturated PAs are further divided into retronecine (RET), heliotridine (HEL), and otonecine (OTO) types (Figure 1B). OTO-type PAs include clivorine and senkirkine, while RET-type PAs include retrorsine and senecionine. HEL-type PAs include heliotrine and lasiocarpine (Figure 1C). Additionally, nitrogen atoms on the necine moiety can be oxidized to form *N*-oxides, which coexist with PAs in most plants [4]. However, otonecine-type PAs are not able to form a corresponding PA *N*-oxide due to the methylated nitrogen in the necine base core structure. Saturated PAs generally exhibit low or no toxicity, while 1,2-unsaturated PAs are of great concern due to their hepatotoxic, carcinogenic, and genotoxic properties [8].

After being absorbed in the small intestine, PAs are transferred to the liver, where they are metabolized by cytochrome P450 enzymes (CYP450) to form active primary metabolites called dehydropyrizidine alkaloids (DHPAs), which are then subsequently hydrolyzed to form dihydropyran derivatives (DHPs) (Figure 1D). DHPAs and DHPs have a strong electrophilicity and can quickly interact with macromolecules in cells, including DNA and proteins, forming pyrrole–DNA adducts, pyrrole–protein adducts, protein–DNA cross-links and protein–protein cross-links [12]. On the other hand, PAs can be hydrolyzed by nonspecific esterase enzymes into necine and necic acid. These necines and necic acids are non-toxic and can bind to polar molecules and be excreted in the urine (Figure 1D). Furthermore, recent studies have shown that PANOs can also cause hepatotoxicity, but the hepatotoxicity in humans is much lower than that of their corresponding PAs [13]. The metabolites of PANOs are generally non-toxic and are excreted in the urine, but excessive amounts of these metabolites can be transformed into toxic epoxides, damaging cellular functions [14]. Studies have shown that in rodents, PAs are primarily metabolically activated by the CYP3A and CYP2B subfamilies. CYP3A4 is involved in the metabolic activation of PAs in humans [15]. The metabolic activation of OTO-type PAs by CYP3A4 is greater than that of RET-type and HEL-type PAs. This results in the formation of more DNA or protein adducts and, therefore, a higher level of toxicity than RET- and HEL-type PAs [16].

## 3. Status of PA Levels in Foods

Recently, several authors have extensively reviewed the food safety issues, toxicity, and risk of PAs [1,7,12]. The most relevant PAs occurring in different food products from 2011 to 2022 are included in Table 1. In fact, herbal teas, honey, food supplements, and spices are the main food items likely to be contaminated with high levels of PAs/PANOs. PAs have also been found in dairy products, meat, beans, and other crops [17,18]. Spices are widely used for culinary flavoring and medicinal purposes. In Europe, the highest levels of PAs in spices were found in cumin and fennel, with levels of 8515 µg/kg and 1653.1 µg/kg, respectively, which were attributed to contamination with seeds of other plants containing high levels of PAs, including *Heliotropium* sp. [19,20]. According to the detection rate of different structural types of PAs, HEL-type europine, heliotrine, and lasiocarpine are the three major PAs most commonly found in spices [20]. The presence of PAs in spices may be related to contamination from PA-producing plants at the cultivation site during harvest or processing [21,22].

Honey is a commonly detected food item associated with high PA levels [23]. The level of PAs in honey varies among different regions. It has previously been reported that honey from Ghana and Ethiopia contains PA levels of 283 µg/kg and 323.4 µg/kg, respectively, which are greater than those of honey from Poland and Germany, which have PA levels of 2.9 µg/kg and 6.1 µg/kg, respectively [17,21,24,25]. In Brazilian honey, senecionine *N*-oxide was detected in 92.3% of 92 commercial honey samples, which is the highest detection rate of PAs, with a maximum concentration of 248 µg/kg [26]. Honey samples from Ethiopia had the highest percentage of lycopsamine detection, with a 100% percentage of positive samples [24]. It is possible that the different PA levels and types in honey are due to the different climates, as studies have shown that the species of *Camellia sinensis* more easily spread and grow than other species in tropical or subtropical regions [27]. OTO-type senkirkine and clivorine are most common in honey [28]. Bee pollen is a mixture of pollen, nectar, and bee saliva that is rich in essential nutrients and bioactive substances and has antioxidant, anti-inflammatory, and antimicrobial activities. The consumption of bee pollen as a food supplement and health product has increased in recent years. The maximum concentration of PAs in pollen-based food additives, pollen, and pollen products set by the European Union (EU) for 2020/2040 (EU, 2020) is 500 µg/kg. Therefore, monitoring PAs in pollen products is necessary to ensure consumer safety. Kath et al. (2019) reported that echivulgarine and its *N*-oxide are the major PA types in pollen from *Echium vulgare* [29]. The concentration of total PAs in bee pollen was approximately one to two orders of magnitude greater than that in honey. It is likely that bee pollen contributes a large number of PAs to honey.

In addition to spices and honey, teas and herbal teas are often contaminated with PAs. Several countries, including Germany, Switzerland, Ireland, Spain, France, and China, have conducted extensive monitoring of PA levels in herbal teas. Bodi et al. (2014) analyzed 247 samples of herbal teas commercially available in Berlin and reported that the total PA concentration could reach 5647 µg/kg. Among the types of herbal teas, black tea, green tea, and Pu’er tea had relatively low PA concentrations, while rooibos tea and melissa tea had higher PA levels, with average levels of 1856.4 µg/kg and 649.6 µg/kg, respectively [21]. RET-type retrorsine and senecionine are most common in rooibos [21]. Bundesinstitut für Risikobewertung (BfR) performed a risk assessment of PAs in (herbal) teas and reported that long-term exposure to herbal teas from specific brands could potentially increase the risk to human health [30]. Switzerland and Ireland also detected PA levels in commercially available herbal teas, with 34.3% of herbal teas in Switzerland containing one or more types of PAs [31], while digestive aid tea in Ireland had a PA content of 1733 µg/kg [32]. The main source of contamination from plant-derived products (spices and tea) is thought to be the incidental coharvesting of PA-containing weeds [33,34]. It has also been proposed that PAs/PANO occur via changes in the soil [35] and via cross-contamination and transfer of PA in plant-based foods [17,36].

Mulder et al. (2018) investigated the levels of PAs in 1105 animal- and plant-derived foods in six European countries. The detection rate and PA levels in animal-derived foods were lower than those in plant-derived foods. Among 746 animal-derived foods, trace amounts of PAs were detected in eggs, milk, and goats, with PA levels of 0.12 µg/kg, 0.17 µg/kg, and 0.11 µg/kg, respectively [18]. Previous studies have shown that PAs in milk are derived mainly from PA-contaminated feed [37], and the ruminal liquids in cows can metabolize and eliminate most PAs [38]. For the plant-derived foods, among the 359 herbal teas, 92% of the samples tested positive for PAs, with an average level of 460 µg/kg. In addition, PAs were also detected in cereal crops such as wheat and maize, with the highest levels reaching 320 µg/kg and 302 µg/kg, respectively [8,17]. Overall, among these types of foods, herbal teas contain the highest level of PAs, followed by spices and honey. The most commonly detected PAs are seneciphylline *N*-oxide, intermedine, and retrorsine *N*-oxide.

**Table 1:** Levels of PAs (µg/kg) detected in various food products originating from different countries.

Type of Food	Number of Detected PAs/PANOs	Top Three Abundant PAs/PANOs	Concentration of Total PAs (Average or Range)	Country	Reference
Rosemary	21	Lasiocarpine, senecivernine *N*-oxide, europine *N*-oxide	253 ± 26	Spain	[19]
Basil leaves			335 ± 29		
Thyme leaf			553 ± 48		
Provence mixed herbs (calendula, rosemary, basil, oregano, etc.)			258 ± 18	France	
Cumin	21	Europine *N*-oxide, heliotrine *N*-oxide, lasiocarpine *N*-oxide	8515.0	Belgium	[20]
Fennel			1653.1		
Melissa tea	17	Seneciphylline-*N*-oxide, retrorsine-*N*-oxide, intermedine	649.6	Germany	[21]
Fennel tea			51.6		
Chamomile			439.7		
Peppermint tea			134.2		
Green tea			109		
Black tea			255.9		
Rooibos			1856.4		
Fennel tea	9	Seneciphylline, senecionine, retrorsine	ND	Switzerland	[31]
Peppermint tea			1.0		
Chamomile			1.9		
Green tea			ND		
Black tea			ND		
Rooibos			1.5		
Black tea	14	Crotaline *N*-oxide, senecionine *N*-oxide, seneciphylline *N*-oxide	19	Ireland	[32]
Oolong tea			ND		
Green tea			ND		
Organic Pu’er tea			ND		
Digestive tea			1733		
Camomile and spearmint tea			1438		
Black tea	14	Jacobine, jacobine-*N*-oxide, seneciphylline	ND-1.91	China	[33]
Green tea			ND-14.3		
Dark tea			ND-151.3		
Chrysanthemum			ND-5.2		
Mixed herbal tea	27	Echimidine, enchinatine *N*-oxide, intermedine	5.8–215	Latvia	[39]
Honey	16	Monocrotaline, echimidine, lycopsamine	0.04–288.1	China	[40]
	17	Lycopsamine, lycopsamine *N*-oxide, monocrotaline	1.5–323.4	Ethiopia	[24]
	27	Echimidine, lycopsamine, senecionine	0.14–74	Latvia	[39]
	10	Echimidine, lycopsamine, intermedine	2.9	Poland	[25]
	17	Seneciphylline-*N*-oxide, retrorsine-*N*-oxide, intermedine	6.1	Austria	[21]
	Total PAs	--	283	Ghana	[41]
	8	Senecionine, senecionine *N*-oxide, monocrotaline	50.5	Brazil	[26]
	Total PAs	--	105	Uruguay	[42]
			53–76	Central and South American countries	
			8	Guatemala	
Bee pollen	18	Echivulgarine, echivulgarine *N*-oxide europine	576	Switzerland	[29]
	17	Echimidine, echimidine *N*-oxide, senecionine	142–3356	Italy	[43]
Cow milk	28	Senecionine, seneciphylline, retrorsine	0.17	Germany, The Netherlands, Spain, France, Italy and Greece	[18]
Goat milk			0.11		
Cheese			ND		
Fresh egg			0.12		
Maize (*Zea mays* L.)	Total PAs	--	0.9–2.0	The Volta region of Ghana	[17]
Wheat	67	Seneciphylline, seneciphylline *N*-oxide, senecionine	0–320	The Netherlands	[8]
Corn			0–302		
Millet			0–302		
Rapeseed			9–308		
Pea			16–315		
Carrot			0–302		

Note: ND (not detected).

## 4. PAs Extraction and Detection Methods

In recent years, the technologies used to detect PAs in complex matrices have developed from qualitative analysis to quantitative analysis of trace levels, including high-performance liquid chromatography (HPLC) coupled with diode array detection (DAD) [44], direct analysis in real-time coupled with mass spectrometry (DART-MS) [45], micellar electrokinetic chromatography with organic modifiers [46], enzyme-linked immunosorbent assays [47], capillary electrophoresis [48], gas chromatography coupled to mass spectrometry (GC–MS) [49], high-performance liquid chromatography (HPLC) coupled to mass spectrometry (MS) [18], HPLC-ion mobility-quadrupole time of flight mass spectrometry (IM-QTOF MS) [50], and high-resolution–tandem mass spectrometry (HRMS) [51]. In view of the high sensitivity and selectivity of GC–MS and HPLCMS/MS, EFSA has suggested the use of these two analytical techniques for the determination of PAs [8]. Kowalczyk et al. (2018) converted all the 1,2-unsaturated alkaloids into the necin backbone (RET-type PAs) with LiAlH_4_ and derivatized them with heptafluorobutyric anhydride (HFBA). Using this method, 1,2-unsaturated PAs were detected in honey by GC–MS, the recovery of which ranged from 73.1 to 93.6%, and the limit of quantification (LOQ) was determined to be 1 µg/kg [49]. The same method was subsequently used to detect PAs in other plant extract substrates [52]. Moreover, PAs were not detected by GC since they do not volatilize at the temperatures required to perform the analysis [53]. HPLC can detect PANOS, and sample preparation is easier and faster than GC preparation since HPLC or UPLC do not require derivatization of PAs/PANO. Table 2 summarizes the different analytical strategies carried out for PAs/PANOs in different food matrices.

The salting-out assisted liquid–liquid extraction (SALLE), liquid–liquid extraction (LLE), or solid–liquid extraction (SLE) methods, in combination with a purification step using solid-phase extraction (SPE), have been used for the simultaneous extraction of PAs/PANOs from spices, herbal teas, and honey, which are food matrices with high concentrations of PAs and PANOs. Due to the high polarity of PAs/PANOs, acidified aqueous solutions or polar organic solvents are usually used for the simultaneous extraction of PAs/PANOs from samples [54]. For honey, a rudimentary extraction of PAs/PANOs was performed based on LLE or SALLE with an acidic aqueous solution (H_2_SO_4_, 0.05 M). For herbal teas, the extraction method was performed using an immersion method with boiling water [18], which can simulate a more realistic exposure scenario. Furthermore, hydrochloric acid, acidified acetonitrile, formic acid (FA), and dichloromethane were also used as extraction solvents. For high-fat foods, such as milk, yogurt, cheese, meat, and eggs, it is usually necessary to first separate the fat components with dichloromethane and hexane [18]. However, the operation of LLE or SALLE is complicated; PAs and PANOs cannot be separated and purified at the same time. In addition, PA loss is too high to meet the requirements of trace analysis of PAs. Thus, these methods are rarely used in isolation.

It is usually necessary to purify sample extracts before analysis due to the complexity of food matrices. SPE is currently the most widely used method for purifying PAs in spices, herbal teas, honey, eggs, and meat. Sample enrichment and purification can be completed simultaneously via SPE. Strong cation exchange (SCX) sorbents are suitable for extracting and purifying PAs/PANOs from food samples, followed by reversed-phase sorbents (mainly based on octadecylsilane ligands (C18)). Bodi et al. (2014) developed a liquid chromatography–tandem mass spectrometry (LC–MS/MS) method with C18 or SCX SPE for the detection of PAs in 274 tea and 87 honey samples. The results showed excellent mean recovery rates ranging from 72% to 122% for fennel and mixed herbal and rooibos teas and slightly lower recovery rates ranging from 45% to 98% for chamomile and black teas by C18 SPE. Moreover, recovery rates ranging from 66% to 96% in cornflower and lavender honey and slightly lower recovery rates of 55–91% in rapeseed honey were determined by SCX SPE [21]. The SPE method, as the most commonly used pretreatment technology, can be used for the determination of PA traces in complex substrates and provides important support for the establishment of PA trace analysis methods in food products. The quick, easy, cheap, effective, rugged, and safe (QuEChERS) is a new rapid pretreatment method for PA testing that simultaneously extracts and purifies samples [55]. The solvents used were LLE and SALLE, and the clean-up sorbents included anhydrous magnesium sulfate (MgSO_4_), sodium chloride (NaCl), and primary-secondary amine (PSA). A QuEChERS-based extraction procedure involving acetonitrile, NaCl, MgSO_4_, sodium citrate, and sodium citrate tribasic clean-up sorbents has been applied for the extraction and analysis of PAs/PANOs in honey and oregano [39,56]. Izcara et al. (2020) presented the development of a miniaturized strategy based on the QuEChERS method combined with UHPLC–MS/MS for the determination of 21 PAs suggested by the European Food Safety Authority. The method showed good performance when using small amounts of sample (0.2 g), organic solvents (1000 µL), clean-up sorbents (175 mg), and partitioning salts (0.65 g). The analytical method was validated via the analysis of 23 oregano samples, with overall average recoveries ranging from 77 to 96% [56]. Kaczynski et al. (2020) adopted an ultrasonic-assisted QuEChERS method to extract and purify PAs from herbal medicine. The study showed that the use of PSA, C18, graphitized carbon black, and MgSO_4_ adsorbents reduced the recovery rate of PAs, which may have been caused by the adsorption of weakly acidic PAs. However, the use of graphene results in good clean-up efficiencies without reducing the recovery (61–128%) of PAs/PANOs. This method was operationally simple and efficient and has been successfully validated for the analysis of PAs/PANOs in peppermint, chamomile, nettle, and linden teas [57].

**Table 2:** Analytical strategies for accessing PAs/PANOs in different types of foods.

Foods	Number of PAs/PANOs	Sample Preparation	Analysis	LOD/LOQ	Recoveries	Ref.
Thyme, oregano, basil, etc.	7-O-acetylintermedine, echimidine, jacobine, etc., 44 PA/PANO	SLE with 0.05 M H_2_SO_4_ followed by SCX-SPE	HPLC-TQ-MS/MS Column: C18	LOD: 0.1–2.6 μg/kg	50–119%	[34]
Oregano	Lasiocarpine, lasiocarpine *N*-oxide, europine, etc., 21 PA/PANO	QuEChERS	UHPLC-IT-MS/MS Column: Polar C18	LOQ: 0.5–25.0 μg/kg	77–96%	[56]
Sorghum, oregano, and mixed herbal tea	Lycopsamine, echinatine, indicine, etc., 33 PAs/PANOs	SLE with methanol containing 0.4% FA followed by reversed phase SPE	RP-U-HPLC-MS/MS Column: Luna Omega C18	LOQ: 0.5–2.0 μg/kg (sorghum); LOQ: 1.0–5.0 μg/kg (oregano); LOQ: 1.0–10.0 μg/kg mixed herbal tea	Sorghum (82– 115%), oregano (80–106%), and mixed herbal tea (78–117%) for the 50 μg kg^−1^ spiking level	[58]
*Echium plantagineum* L. honey	Echimidine and echimidine *N*-oxide	LLE with 0.05 M H_2_SO_4_ followed by SCX-SPE.	HPLC-DAD	--	--	[44]
Monofloral and multifloral honey	Erucifoline, echimidine, echimidine *N*-oxide, etc., 28 PAs/PANOs	SALLE with acid aqueous solution	UHPLC-HRMS/MS Column: Polar C18	LOQ: 0.1–2.1 μg/kg	63.3–103.9%	[51]
Honey from the Latvian market	lycopsamine *N*-oxide, retrorsine *N*-oxide, retrorsine, etc., 30 PAs/PANOs	QuEChERS (acetonitrile containing 1% FA, MgSO_4_, etc.)	Nano-LC-Orbitrap MS Column:C18	LOQ: 0.05–2.5 μg/kg	--	[39]
Honey	1,2-unsaturated retronecine-type PAs	LLE with 0.15 M HCL and addition of zinc, followed by MCX-SPE; derivatization with HFBA	GC-MS/MS Column: DB-5MS	LOQ:1 μg/kg	73.1–93.6%	[49]
Bee pollen	Lycopsamine, senecionine, seneciphylline *N*-oxides, etc., 17 PAs/PANOs	--	Near-infrared (NIR) spectroscopy	LOQ:0.4 μg/kg	--	[59]
Chamomile tea	Europine, europine *N*-oxide, heliotrine, etc., 21 PAs/PANOs	C18 μ-SPEed cartridge with two aspiration dispense cycles of 100 μL of MeOH followed by 100 μL of water	UHPLC-IT-MS/MS Column: Luna Omega Polar C18 column	--	76–101%	[36]
Black tea and green tea	Echimidine, echimidine *N*-oxide, erucifoline, etc., 28 PAs/PANOs	SALLE with acid aqueous solution	UHPLC-HRMS/MS Column: C18	LOQ: 1–12 μg/kg	63.9–116.9%	[51]
Fresh tea	Retrorsine, senecionine, jacobine, etc., 15 PAs/PANOs	LLE with 0.1 M H_2_SO_4_ followed by PCX SPE	UHPLC–MS/MS Column: HSS T3	LOQ:1–5 μg/kg	67.0–111.9%	[60]
Herbal tea	Riddelliine, Riddelliine *N*-oxide, seneciphylline, etc., 34 Pas/PANOs	infusion with boiling water followed by C18-SPE	UHPLC-TQ-MS/MS Column: C18	LOD:0.2–3.8 μg/kg	45–122%	[18]
Tea from the Latvian market	Lycopsamine, lycopsamine *N*-oxide, retrorsine, etc., 30 PAs/PANOs	QuEChERS (acetonitrile containing 1% FA, MgSO_4_, etc.)	Nano-LC-MS-Orbitrap MS Column: C18	LOQ: 0.5–20 μg/kg	--	[39]
Milk				LOQ: 0.5–20 μg/kg	--	
Fresh milk	Senecionine, senkirkine, seneciphylline, etc., 6 PAs/PANOs	LLE with 0.5% FA and dichloromethane	DART-IT-MS	LOQ:1.83–2.82 ng/mL	89–112%	[45]
Milk, yogurt, cheese	Riddelliine, riddelliine *N*-oxide, seneciphylline, etc., 34 PAs/PANOs	LLE or SLE with hexane containing 0.2% FA followed by reversed-phase SPE	UHPLC-TQ-MS/MS Column: C18	LOD: 0.03–0.05 μg/L (milk and yogurt); LOD: 0.05–0.15 μg/kg (cheese)	74–107%	[18]
Eggs, pork meat, beef, liver				LOD: 0.05–0.15 μg/kg (egg, pork, and meat); LOD: 0.1–0.25 μg/kg (beef and liver)	Egg (56–103%); meat product (63–91%)	
Eggs and meat	Senecionine, seneciphylline, riddelliine, etc., 51 PAs/PANOs	SLE with hexane containing 0.2% FA followed by reversed phase SPE	UHPLC-TQ-MS/MS Column: C18	LOQ:0.1–1 μg/kg	--	[61]
Maize	1,2-unsaturated RET/HEL-type PAs	SLE with 0.05 M H_2_SO_4_ followed by SCX-SPE	HPLC-QTRAP-MS/MS Column: C12	--	--	[17]
Herb (atractylodis rhizoma, chrysanthemi flos, leonuri herba, gastrodiae rhizoma, glycyrrhizae radix)	Retrorsine, Senkirkine, Lycopsamine *N*-oxide, etc., 28 PAs	0.05 M H_2_SO_4_ in 50% MeOH followed by MCX-SPE	LC–MS/MS Column:Shim-pack GIST-C18	LOQ: 0.1–6.5 μg/kg (Atractylodis Rhizoma); LOQ: 0.1–10.1 μg/kg (Chrysanthmi Flos); LOQ: 0.1–5.5 μg/kg (Leonuri Herba); LOQ: 0.1–9.1 μg/kg (Gastrodiae Rhizoma); LOQ: 0.1–10.5 μg/kg (Glycyrrhizae Radix)	Atractylodis Rhizoma (72.5–123.7%); Chrysanthmi Flos (70.6–151.7%), Leonuri Herba (80.6–130.9%), Gastrodiae Rhizoma (70.3–122.9%), Glycyrrhizae Radix (67.1–106.9%)	[62]
Herb (peppermint, chamomile, nettle, and linden)	Echimidine, erucifolin, heliotrine, etc., 30 PAs/PANOs	QuEChERS (acetonitrile containing 1% FA, followed by graphene to clean up)	LC–MS/MS Column: Hypersil Gold	LOD: 0.05–0.15 μg/kg (egg, pork, and meat); LOD: 0.1–0.25 μg/kg (beef and liver)	61–128%	[57]

## 5. Toxic Effects of PAs

### 5.1. Acute Toxicity

Since the early 19th century, it has been observed that livestock that consume plants belonging to the genera *Heliotropium*, *Senecio*, or *Crotalaria* experience slow emaciation and weakness, and has autopsies have revealed hepatocyte necrosis [63,64]. Acute poisoning by PAs can significantly affect the liver, leading to acute veno-occlusive disease characterized by hepatomegaly, hemorrhage, ascites, and even death in severe cases [1]. It has been reported that a 6-month-old female infant was diagnosed with hepatic veno-occlusive disease (HVOD) after the ingestion of PAs at approximately 0.8 to 1.7 mg/kg body weight (b.w.) per day for 2 weeks [1]. Similarly, a 2-month-old male infant ingesting 3 mg/kg (b.w.) PAs per day died after approximately 4 days [65]. Based on epidemiological data, the EFSA Panel on Contaminants in the Food Chain estimated that the daily intake of PAs ranging from 1 to 3 mg/kg (b.w.) per day for 4 to 14 days can cause acute toxicity [8]. It was shown that 7R-configured macrocyclic diesters of PAs, including retrorsine, seneciphylline, and senecionine, constitute the most potent group causing acute toxicity [66,67,68]. The 7S-synthesized Pas heliotrine and lasiocarpine show acute toxic effects similar to macrocyclic diesters [66]. Compared with 7S-heliotrine, 7S-lasiocarpine, and 7R-configured macrocyclic diesters of PA, 7R-echimidine, 7S-heliotrine, 7R-indicine, and 7R-intermedine had lower acute toxic effects [69]. Furthermore, the acute toxicity of PANOs generally appears to be lower than that of their parent PAs [13].

### 5.2. Cytotoxicity

The cytotoxicity induced by Pas is also structurally dependent. Li et al. (2013) evaluated the cytotoxicity of four PAs, namely, seneciphylline, senecionine, retrorsine, and riddelliine, on HepG2 cells using MTT and bromodeoxyuridine (BrdU) incorporation assays. MTT results showed that the IC_20_ value of senecionine was 0.66 mM, which was 2.4, 1.9, and 2.1 times greater than those of retrorsine, senecionine, and riddelliine, respectively. Moreover, the BrdU assay showed similar results [70]. Reuel A Field et al. (2015) assessed the effects of 11 PAs on cell morphology, mitochondrial function, and lactate dehydrogenase (LDH) activity in CRL-2118 chicken hepatoma cells. MTT and LDH assays revealed that lasiocarpine had the greatest cytotoxicity, followed by riddelliine, heliotrine, seneciphylline, and senecionine. The cytotoxic effects of these PAs are characterized by significant cell swelling and vacuoles. On the other hand, the cytotoxicities of riddelliine *N*-oxide, senecionine *N*-oxide, and heliotrine *N*-oxide were lower than those of monocrotaline, intermedine, and lycopsamine [71]. In general, the cytotoxicity caused by macrocyclic diesters (RET and HEL types) with cyclic and acyclic ester structures, such as lasiocarpine, seneciphylline, and riddelliine, was greater than that caused by monoesters (heliotrine, lycopsamine, and intermedine) and PANOs. Current studies on the cellular toxicity mechanisms of PAs have focused mainly on oxidative stress and apoptosis, with oxidative stress being the primary cause of cytotoxicity. Several studies revealed that DHPAs and DHP not only bind to DNA and proteins to form adducts but also bind to glutathione (GSH) to form adducts. When GSH is depleted and not supplied in a timely manner, it leads to oxidative stress and consequent cytotoxicity [72]. Previous studies have shown that exposure of rat hepatocytes to adonifoline, monocrotaline, and clivorine significantly reduces intracellular GSH levels, and the activities of glutathione peroxidase (GSH-Px), glutathione reductase (GR), and glutathione S-transferase (GST) also significantly decrease [72,73]. In addition, apoptosis is another crucial factor contributing to cytotoxicity. Several studies have demonstrated that PAs can cause hepatotoxicity by activating apoptosis [74]. Clivorine treatment of L-02 cells resulted in a significant increase in intracellular caspase-3 enzyme activity and increased expression levels of cleaved PARP and cleaved caspase-3 proteins [75,76]. Furthermore, the induction of apoptosis and subsequent cytotoxicity can also be attributed to the downregulation of the antiapoptotic factor Bcl-xl and the upregulation of Fas expression [75,77]. Table 3 summarizes the IC_50_ and IC_20_ values for cytotoxicity caused by different types of PAs.

### 5.3. Genotoxicity and Carcinogenicity

Animal experiments have shown that 1,2-unsaturated PAs can cause hepatocarcinoma in rodents [79,80]. The main reason is that DHPAs and DHPs generated by 1,2-unsaturated PAs in the liver bind to DNA to form adducts, resulting in abnormal biological processes and genotoxicity, which are also considered to be the main reasons for the carcinogenic effects of PAs [4,81]. In recent years, a series of in vitro assays have demonstrated that most 1,2-unsaturated PAs are genotoxic [81]. Williams et al. (1980) developed a quantitative detection method using radioactive precursors to detect DNA damage, and the results showed that lasiocarpine and riddelliine can induce nonprogrammed DNA synthesis in primary rat hepatocytes [82]. Monocrotaline, riddelliine, senecionine, and seneciphylline can induce DNA repair and HGPRT gene mutation in rat hepatocytes [83].

The number of DNA adducts induced by PAs with different structures results in differences in genotoxic and carcinogenic potentials. Xia et al. (2013) quantitatively analyzed the DNA adducts of nine PAs, including lasiocarpine, riddelliine, retrorsine, retronecine, heliotrine, clivorine, monocrotaline, senkirkine, and lycopsamine, in rat livers. The results showed that the levels of DNA adducts formed by macrocyclic diesters (retrorsine, riddelliine, and monocrotaline) and acyclic diesters (lasiocarpine) were much greater than those formed by monoester (glycosamine), HEL-type (heliotrine), and OTO-type clivorine (clivorine and senkirkine) [84]. Louisse et al. (2019) assessed the genotoxicity of 37 PAs in HepaRG cells using a γH2AX assay, and the results showed that the genotoxicity of cyclic diester and macrocyclic diester PAs was greater than that of monoester PAs and PANOs [85]. In general, the genotoxicity of PAs is characterized by higher levels of acyclic and macrocyclic diesters than monesters, and RET- and HEL-type PAs generally exhibit greater toxicity than OTO-type PAs.

## 6. Risk Assessment of PAs

Currently, there are limited toxicological data from animal studies and epidemiological data in humans, leading to uncertainty in dietary exposure assessment of PAs [3]. Risk assessment for chemicals typically relies on toxicological data obtained from animal studies. However, there are differences in biological responses between mammals and humans, which means that data from animal experiments cannot fully represent human responses [86]. To date, the best method for conducting risk assessments of PAs is the margin of exposure (MOE) approach [87]. The MOE is a dimensionless ratio between the toxicity threshold obtained from epidemiological and tumor incidence data and the estimated daily intake (EDI) in the human population [87,88], with the toxicological threshold obtained using the benchmark dose (BMD) method [88]. In addition, the 95% lower confidence interval (CL) of the BMD (BMDL) value was further applied when considering statistical uncertainty [88]. BMD and BMDL values are used to define the incidence of tumors; this parameter is known as the benchmark response (BMR) and includes 1%, 5%, or 10% of the incidence above background tumor rates [89]. Generally, it is preferable to use the BMDL_10_ to calculate the MOE since the use of a lower incidence rate increases uncertainty, as a 1% or 5% incidence rate may exceed the experimentally observed incidence rate [88]. Obtaining BMDL_10_ values requires a 2-year animal carcinogenicity study. Currently, only lasiocarpine and riddelliine have BMDL_10_ values derived from animal experiments. However, only the BMDL_10_ value of riddelliine was used for risk assessment. This is because EFSA recalculated the BMDL_10_ values for lasiocarpine and riddelliine based on 2-year carcinogenicity data more recently, and the results showed that the previously calculated BMDL_10_ of lasiocarpine was affected by a high degree of uncertainty [8]. The critical value of the ratio of the BMDL_10_ to the EDI was set at 10,000. A value less than 10,000 suggests that long-term intake of PAs from certain foods may pose a potential risk to human health. The value of 10,000 contains three uncertainty factors, including a factor of 100 for species differences and human variability in kinetics and dynamics, a factor of 10 for variability in cell cycle control and human DNA repair, and a factor of 10 because BMDL_10_ is not an observed adverse effect level (NOAEL) [90]. Compared to the hazard index method, the MOE takes into account more uncertainty factors, making it more suitable for evaluating substances such as PAs, which are genotoxic carcinogens [9,91,92]. Using the MOE method, EFSA and BfR were used to evaluate the risk of PA exposure in food, and the results showed that the MOE for long-term intake of specific herbal tea brands in adults ranged from 789 to 900, indicating a potential risk to human health [30]. Chen et al. (2017) conducted a risk assessment for herbal tea and plant food supplements, and the results showed that consumption of a cup of tea per day over a lifetime would lead to MOE values of less than 10,000 for several types of herbal teas, such as rooibos tea, peppermint tea, and melissa tea, indicating that risk management should be prioritized for these products [93]. For plant food supplements, the MOE values varied considerably among the different types of plant food supplements, ranging from 7900 to approximately 17,500,000. BfR also evaluated dietary exposure in groups frequently consuming herbal tea, and the MOE was 789–4098, suggesting the establishment of reasonable dietary habits and the avoidance of simultaneous intake of PA-containing foods [30]. Table 4 summarizes the MOE values for a variety of food products.

Notably, foods often contain one or more types of PAs [3,8]. When using the MOE approach, the BMDL_10_ values are derived from lasiocarpine or riddelliine, considering their greater hepatotoxicity than other PAs [13], which may lead to an overestimation of the risks. In addition, each type of PA has a different toxic potency, so simply summing the concentrations of all PAs may result in either an overestimation or underestimation of the risks [8]. The relative potency factor (REP) correction serves as an approach for the risk assessment of PAs with a similar mode of action. The potency of each component in a mixture is compared to that of a reference chemical, generating a measure of potency for each component with respect to the toxicity of the index chemical. It is more accurate to perform risk assessments for PAs in foods by correcting each PA concentration in the mixtures using REP factors. Merz and Schrenk (2016) derived interim relative potency factors for PAs, suggesting a factor of 1.0 for cyclic diesters and open-chained diesters with the 7S configuration, 0.3 for monoesters with the 7S configuration, 0.1 for open-chained diesters with the 7R configuration and 0.01 for monoesters with the 7R configuration. For *N*-oxides, we suggest the REP factor of the corresponding PA [94]. Chen et al. (2022) performed a risk assessment of herbal teas containing PAs based on REP factors; results showed that the total PA levels were decreased by REP correction in most of the teas [91]. The use of RPFs does not reduce the levels analyzed in certain foodstuffs, but it only reduces (or enhances) the resulting risk of the PA portfolio determined in the sample.

**Table 4:** The MOE values for the specific type of food products.

Food	MOE Value	References
Herbal tea	3121	[30]
Tea	1872	[30]
Peppermint tea	5400	[93]
Rooibos	4200	[93]
Black tea	6000	[93]
Green tea	6200	[93]
Melissa tea	3800	[93]
Chamomile tea	14,100	[93]
Nettle tea	10,300	[93]
Fennel tea	47,400	[93]
Anise	54,000	[93]
Fennel	1,467,000	[93]
Coriander	14,100	[93]
Nettle	304,000	[93]
Honey	593,000	[8]
Commercial honey in Brazil	5010	[95]
Pollen-based plant food supplements	561,000	[93]
Mixed plant extracts	415,000	[93]

## 7. Challenges in Risk Assessment of PAs

Recently, the EFSA proposed that a set of 17 PAs be monitored in food and feed [8]. Due to the lack of toxicology data on PAs, different countries and organizations have established different regulations on PAs in foods (Table 5). For instance, the Committee on Toxicity of Chemicals in Food, Consumer Products and the Environment (COT) proposed a maximum level limit of 6.4 μg/kg for PAs in honey and set the upper limit of daily intake of PAs at 0.007 μg/kg (b.w.) per day [96]. The Dutch National Institute for Public Health and the Environment (RIVM) proposed a maximum daily intake of 0.1 μg/kg (b.w.) for PAs [97], while BfR recommends a maximum daily intake of 0.007 μg/kg (b.w.) for PAs [30]. The European Medicines Agency (EMA) provides more detailed regulations, suggesting a daily intake of 0.35 μg/kg (b.w.) for adults [98]. This maximum level set by the EMA can be applied only to herbal medicinal products and not to food or feed [98]. Currently, China has not set specific regulations or maximum daily intake of PAs in food. Online sales of PA-containing foods have further complicated matters since consumers are able to buy products globally. The regulatory oversight of PA-containing foods needs to be increased to address these issues and ensure public health. It is imperative that risk assessment criteria be harmonized and that internationally recognized PA-contaminated food compendia be established.

Additionally, the accuracy of PA risk assessment is limited by the absence of toxicological data for the majority of PAs. Conducting animal experiments is time-consuming and costly, leading to an increasing trend of using in vitro materials such as cell cultures or tissues as alternatives to animal testing. From 2011 to 2017, the Organization for Economic Co-operation and Development (OECD) continually updated and published guidelines for in vitro testing methods. In 2013, the Interagency Coordinating Committee on the Validation of Alternative Methods (ICCVAM) in the United States established a strategic blueprint for the development of nonanimal testing methods in toxicology research [100]. In addition, initiatives such as the TOXCAST screening program by the Environmental Protection Agency (EPA) and projects such as AXLR8 in the EU signal a new era in in vitro toxicology research. However, extrapolating in vitro test results to in vivo data often leads to uncertainty due to the lack of knowledge about the absorption, distribution, metabolism, and excretion (ADME) processes of compounds in the body [101]. Therefore, addressing the issue of data extrapolation is a key challenge in food safety assessment research. On one hand, traditional alternative methods for in vitro use have been far improved so that they can allow for more complex testing on systemic and target organ toxicity. On the other hand, computer modeling, such as physiological-based pharmacokinetic (PBK) models, can simulate the ADME processes of substances in the body using a set of mathematical equations, effectively addressing the uncertainty associated with extrapolating from in vitro toxicity data to the in vivo situation in the body [101]. The development of a PBK model is based on physiological and anatomical parameters, physicochemical parameters, and kinetic parameters [102,103]. Via simultaneously solving these PBK equations, the outcome can indicate, for instance, the time- and dose-dependent changes in concentrations of a compound or its relevant metabolite(s) involved in the metabolic process as well as dynamics in blood and tissues. In addition, PBK modeling is a valuable tool for predicting species-specific and relevant exposures to help determine safe external levels of chemicals based on internal doses to laboratory animals, humans, and target organs of organisms used in environmental risk assessments [104]. Allemang et al. (2018) calculated the REP factors for 15 PAs using a micronuclei formation assay in HepaRG cells [13]. Lasiocarpine and riddelliine have relatively high potency, but the in vitro genotoxicity of lasiocarpine was 6 times greater than that of riddelliine in a micronucleus study [13,94]. Chen et al. (2019) conducted in vitro genotoxicity testing using the γH2AX assay for lasiocarpine and riddelliine. The results showed that the in vitro genotoxicity of lasiocarpine was 3.5 times greater than that of riddelliine [105]. However, when using a PBK model to extrapolate the in vitro toxicity data of lasiocarpine and riddelliine, the in vivo genotoxicity of riddelliine was 2.6 times greater than that of lasiocarpine, and these results are consistent with those of animal studies [105]. This indicates that defining REP values based on in vitro data without taking possible differences in toxicokinetics into account may result in inaccurate values. Preferably, the REP values used in the MOE-based risk assessment of combined exposure to PAs should be derived from in vivo carcinogenicity potencies. To date, PBK models have been successfully used for predicting the in vivo toxicity of chemicals present in food, such as reproductive and developmental toxicity [106], nephrotoxicity [107], and endocrine toxicity [108], and are increasingly being recommended by the US EPA and EFSA [109]. To better refine the REP values, data on PA toxicity could be combined with PBK modeling. Such an in vitro-in silico method could be used for different PAs, especially the 17 PAs that have been identified by EFSA as relevant for exposure via feed and food [8]. PBK modeling-based reverse dosimetry for quantitative in vitro to in vivo extrapolation (QIVIVE) can be used to consider the toxicokinetics of PAs and estimate the REP values for PAs closer to their in vivo difference, which will allow for more accurate risk assessment of PAs.

## 8. Conclusions

Here, we summarized the major PAs present in various food products. In terms of PA contamination in foods, PAs are most abundant in herbal teas, spices, and honey products (Table 1). In herbal teas, seneciphylline *N*-oxide and retrorsine *N*-oxide are predominant among the detected PAs. In contrast, the most abundant PAs in spices are europine *N*-oxide, heliotrine *N*-oxide, and lasiocarpine, while echimidine and lycopsamine are the top two PAs in honey. Notably, spices generally contain significant levels of lasiocarpine or its *N*-oxide form, which have been considered the most potent PA that can cause carcinogenic effects. With the continuous detection of PAs in plants and plant-derived foods, the potential risks of PAs to human health have attracted increasing concern in the area of food quality and safety. Due to the increase in food alerts noted in recent years, regulations regarding maximum concentration levels of PAs in food products have been set or proposed by multiple organizations. In general, food products that are likely to be contaminated with PAs are listed, including teas, herbal teas, honey, pollen, aromatic herbs, and some spices and food supplements. Currently, the WHO, BfR, EMA, and UK Toxicity Committee have established acceptable daily intake for total PAs. For example, the European Union officially announced a total limit of 150 μg/kg for PAs in teas and flavored teas (CR (EU) 2023/915 amending regulation (EC) 1881/2006). China has not yet set relevant limits for PAs in any type of food product, but increasing amounts of studies aiming for risk assessment of PAs have been performed in China in recent years [91,110]. Establishment and modification of acceptable daily intake for PAs in foods are important for public health and international trade and may benefit from in vitro alternative toxicology, such as PBK modeling and MOE approach. To strengthen the risk management for PA contamination in foods, the international regulatory community may generate a list of foods with known safety issues and update the list regularly, as is the case for the outline produced by the EFSA. In addition, manufacturers should be requested to supply ingredient information, and highly sensitive analytical methods are also required to monitor PA concentrations during the sampling and production process. These quality control practices will assist in risk assessment and ensure the safety of foods.

## Figures and Tables

**Table 3:** IC_50_ or IC_20_ values for different types of PAs.

PAs	Cell Line	Exposure Dose (μM)	Exposure Time (h)	IC_50_/IC_20_ (μM)	References
Seneciphylline	HepG2	62.5, 125, 250, 500, 1000	24	660 ^a^	[70]
Clivorine				130 ^a^	
Retrorsine				270 ^a^	
Platyphylline				850 ^a^	
Senecionine				340 ^a^	
Lasiocarpine	CLR-2118	19, 38, 75, 300	24	14 ^b^	[71]
Senecionine	mouse primary hepatocytes	1, 3, 10, 30, 100	48	5.41 ^b^	[78]
Adonifoline				49.91 ^b^	

^a^ IC_20_ ^b^ IC_50_; IC20 values refer to the calculated 20% inhibitory concentrations on cell viability; IC50 values refer to half-maximal inhibitory concentrations on cell viability.

**Table 5:** Summary of the maximum daily intake of PAs.

Institution or Organization	Maximum Daily Intake (µg/kg b.w/day)	References
World Health Organization (WHO)	15	[99]
BFR	0.007	[30]
COT	0.007	[96]
EMA	Adult 0.35 Children 0.007	[98]
RIVM	0.1	[97]

## Data Availability

Not applicable.
